# FDG-PET/CT for oral focus assessment in head and neck cancer patients

**DOI:** 10.1007/s00784-022-04403-2

**Published:** 2022-03-07

**Authors:** Dominic Raphael Schwaninger, Martin Hüllner, Dominique Bichsel, Barbara Giacomelli-Hiestand, Nicole Selina Stutzmann, Panagiotis Balermpas, Silvio Valdec, Bernd Stadlinger

**Affiliations:** 1 Clinic of Cranio-Maxillofacial and Oral Surgery, Center of Dental Medicine, University of Zurich, Plattenstrasse 11, 8032 Zurich, Switzerland; 2 Department of Nuclear Medicine, University Hospital Zurich/University of Zurich, Zurich, Switzerland; 3 Statistical Services, Center of Dental Medicine, University of Zurich, Zurich, Switzerland; 4 Department of Radiation Oncology, University Hospital Zurich, University of Zurich, Zurich, Switzerland

**Keywords:** Positron emission tomography–computed tomography, Panoramic radiography, Periapical radiography, Radiochemotherapy, Oral focus, Head and neck cancer

## Abstract

**Objectives:**

To compare oral and maxillo-mandibular inflammatory foci on standard oral radiographs (OPT, periapical radiograph) with available fluorine-18-labelled fluoro-2-deoxy-D-glucose-positron emission tomography/computed tomography (FDG-PET/CT) data and to discuss whether additional metabolic information derived from FDG-PET/CT can support oral care specialists when performing oral focus examinations.

**Materials and methods:**

Data from 23 patients with head and neck cancer who underwent FDG-PET/CT and panoramic and periapical radiography in close succession before first-line radiotherapy and/or chemotherapy were included in this exploratory retrospective study. Periapical lesions and marginal periodontal inflammation on FDG-PET/CT scans and standard oral radiographs were analysed and compared with regard to metabolic activity on FDG-PET/CT in comparison to recorded clinical symptoms and radiological scores. Additionally, inflammatory maxillo-mandibular pathologies were analysed using FDG-PET/CT.

**Results:**

The maximum standardised uptake value (SUV_max_) in FDG-avid marginal periodontal sites could not be conclusively associated with the radiologically recorded severity of marginal bone loss, but a potential positive correlation was identified. No association was found either between the metabolic activity of periapical lesions and their extent, as recorded on standard oral radiographs, or regarding clinical symptoms (percussion test). Most maxillo-mandibular pathologies did not show increased FDG uptake.

**Conclusions:**

FDG-PET/CT provided additional metabolic information that can help clinicians identify lesions with increased inflammatory activity. The incorporation of available oral FDG-PET/CT findings into the primary oral focus assessment may allow for more accurate oral focus treatment.

**Clinical relevance:**

FDG-PET/CT provides valuable metabolic information for oral care specialists. The detection of inflammatory oral processes using FDG-PET/CT facilitates treatment.

## Introduction

Head and neck cancer (i.e., malignancies of the sinuses, oral cavity, pharynx, and larynx) accounts for a worldwide incidence of more than 650,000 cases and is responsible for approximately 330 000 deaths per year [1]. Patients are mostly diagnosed with head and neck cancer between the ages of 55 and 65 years, with the incidence in men being twofold that in women [2]. Cancer treatment usually involves surgical resection and/or radiotherapy (RT) with or without chemotherapy. Prior to oncologic treatment, patients should undergo oral health screening, including clinical and radiological examinations, to detect potential intraoral inflammatory foci that require immediate treatment [3]. Acute oral disease foci increase the risk of infective complications during and after various oncological treatments such as radiotherapy, myelosuppressive chemotherapy, organ or haematopoietic stem cell transplantation, and long-term immunosuppressive therapy [4, 5]. Recommendations for oral focus assessment facilitate the improvement of pre- and posttreatment dental care in head and neck cancer (HNC) patients scheduled for radio- and chemotherapy [6, 7].

Short-term and long-term oral complications after radiotherapy range from temporary taste dysfunction to osteoradionecrosis ORN [8]. Compared with previous radiotherapy techniques, modern intensity-modulated radiotherapy (IMRT) causes less damage to healthy oral tissues surrounding a tumour without lower-quality treatment outcomes [9].

Regarding the pre-interventional detection of dentoalveolar and maxillomandibular inflammatory foci, it is important to perform both clinical examinations and radiological imaging. Panoramic radiography (OPT) provides an overview of hard oral tissues. This implies advantages owing to its relatively low radiation exposure, good image quality, and widespread availability. OPT was supplemented with periapical radiographs of selected cases. Cone-beam computed tomography serves as a supplementary imaging modality that provides 3D information [10].

Fluorine-18-labelled fluoro-2-deoxy-D-glucose-positron emission tomography/computed tomography (FDG-PET/CT) visualises glucose uptake in tissues, such as primary tumours, metastases, or inflammatory lesions. According to various pertinent guidelines and depending on the primary tumour site and type, FDG-PET/CT imaging may be indicated in patients with head and neck cancer [11, 12]. FDG-PET/CT may be particularly useful in patients with a high pretest likelihood of metastatic disease, such as those with tumours > T2 and/or clinically positive necks. Limited attention has been paid to the visualisation of oral foci using FDG-PET/CT. FDG-PET/CT information may help differentiate between an active periapical infection and metabolically inert periapical granuloma [13]. In this study, the term foci refers to inflammatory foci.

The aims of this study were (1) to analyse the distribution and metabolic activity of periapical radiolucencies, marginal periodontitis, and possibly inflammatory maxillo-mandibular pathologies noted on FDG-PET/CT scans and (2) to compare these with standard oral radiographs. The comparison was performed on an exploratory basis.

## Materials and methods

### Study design and patient selection

This exploratory retrospective study analysed charts and images of HNC patients who underwent FDG-PET/CT, OPT, and periapical radiographs prior to ablative surgery, radiotherapy, and/or chemotherapy between 1 January 2016 and 31 December 2017. All the patients underwent FDG-PET/CT at the Department of Nuclear Medicine, University Hospital, Zurich, Switzerland. Additionally, all patients underwent clinical and radiological oral evaluations at the Clinic of Cranio-Maxillofacial and Oral Surgery, Centre of Dental Medicine, University of Zurich. Additional inclusion criteria for this study were as follows: signed informed consent by patients for the use of their medical data for research and a time interval of no more than 4 weeks between FDG-PET/CT and OPT/periapical radiographs. This study was approved by the local ethics committee of Zurich (no. 2017–01,378). The exclusion criteria were as follows: focal evaluation due to diseases other than HNC; lack of FDG-PET/CT, OPT, or periapical radiographs; temporal spacing of more than 4 weeks between FDG-PET/CT and OPT/periapical radiographs; and low-quality oral radiographs.

In total, 537 oral evaluations were conducted between 1 January 2016 and 31 December 2017. These included evaluations before organ transplantation and evaluations in the context of cardiac, haematological, or oncologic diseases. A total of 23 HNC patients (15 men [65.2%] and 8 women [34.8%]; mean age 68.5, range: 46–92 years]) fulfilled the inclusion criteria. All other patients were excluded as they did not meet one or more inclusion criteria (Table 1). Thus, a total of 418 teeth were analysed for dento-alveolar parameters, and 92 jaw quadrants were analysed for maxillo-mandibular parameters.

**Table 1:**
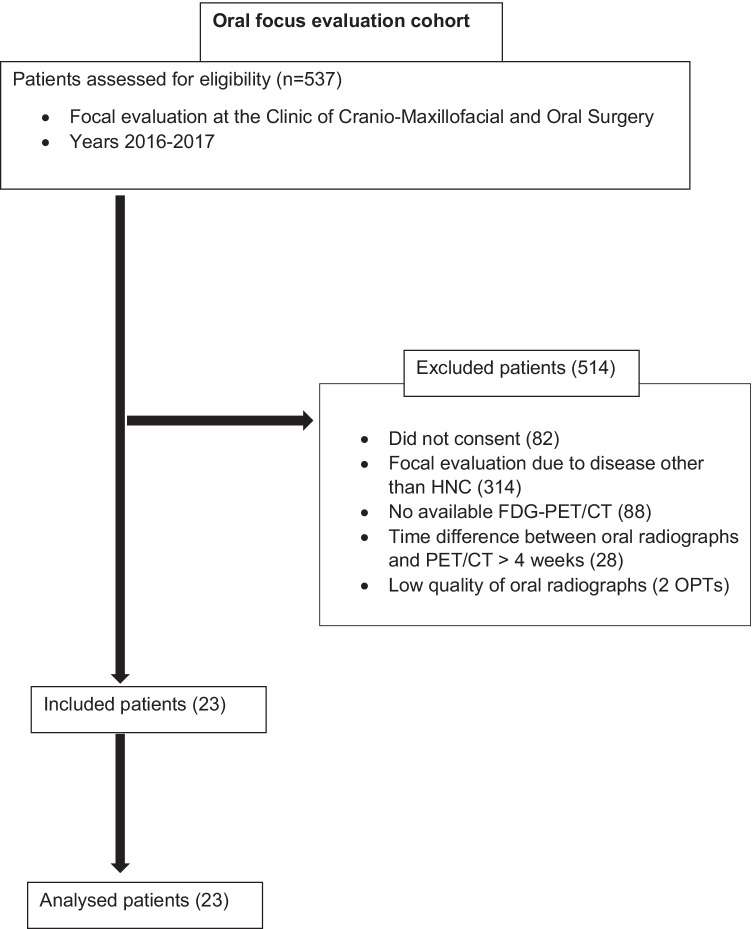
Included/excluded patients. HNC, head and neck cancer; FDG, fluorodeoxyglucose; PET, positron emission tomography; CT, computed tomography

Periapical translucencies were compared between PET/CT and oral radiographic imaging to analyse metabolic activity in different radiological findings.

### Image acquisition

Detailed FDG-PET/CT acquisition protocols have been reported previously [14, 15]. In brief, fasting patients with a blood glucose serum concentration below 12 mmol/l were injected with a standardised dose of 18F-FDG, according to our institution’s protocol. After the injection, the patients rested for one hour in supine position in a warm environment. Scanning was performed using integrated PET/CT systems (Discovery MI PET/CT or Discovery PET/CT 690, GE Healthcare, Waukesha, WI, USA).

OPTs were generated in a standing position, with the head oriented to the Frankfurt plane, using a Cranex 3D (Soredex, KaVo, Biberach, Germany). Periapical radiographs were generated using a Heliodent DS (Dentsply-Sirona, Bensheim, Germany) intraoral X-ray, operating at 60 kV and 7 mA. The parallel technique was used with a focus-patient distance of approximately 21 cm.

### Image analysis

OPTs and periapical radiographs from the focal evaluation time point were analysed. Any blurred radiographic images or OPTs in which tumour structures overlaid areas of interest and thus could not be adequately analysed were excluded.

All periapical radiographs and OPTs were analysed in DICOM format using Synedra Viewer (Synedra, Apollon, Innsbruck, Austria) on a diagnostic monitor (NEC, MDview 243).

All FDG-PET/CT scans were analysed using an Advantage Workstation version 4.6 (GE Healthcare), which enables the viewing of CT, PET, and PET/CT images in overlay mode in three different planes side by side. Foci with abnormally increased FDG uptake in the upper and lower jaw were searched, and their location was recorded. The maximum standardised uptake value (SUV_max_) within the region of interest was assessed using a volume-of-interest tool with an SUV_max_ threshold of 42% and manual adjustment to the individual lesion if necessary. All FDG-PET/CT scans were analysed by a triply board-certified nuclear medicine physician/radiologist/neuroradiologist with 12 years of experience in head and neck imaging.

The OPTs and periapical radiographs were assessed by a dentist (DS) under the supervision of an oral surgeon (DB). In cases of discordance, a decision was made by consulting a third reader (BS). The data were separately collected from both conventional imaging modalities (OPT, periapical radiograph) and fused to a joined dataset, referred to as standard oral radiographs. All imaging modalities (periapical radiography, OPT, and FDG-PET/CT) were analysed in different sessions.

### Radiological data collection

For radiological data collection, the following structures were assessed using FDG-PET/CT and standard oral radiography: 1) periapical lesion, 2) marginal periodontium, 3) fully or partially impacted teeth, 4) non-odontogenic bone lesions, 5) mandibular condylar pathology, and 6) maxillary sinus pathology FDG-PET/CT were further analysed to determine the metabolic activity of these structures. Periapical lesions were radiologically classified according to the periapical index (PAI) [16] into five groups, ranging from 1 (healthy) to 5 (severe, exacerbating apical periodontitis). Periodontal inflammation was assessed based on the marginal bone level. A score of 1–4 was applied [17] according to the marginal periodontitis index (MPI). The estimated physiological bone level was compared with the actual bone level. Bone loss of less than one-third was classified as ‘1’, one-third up to half as ‘2’, half up to two-thirds as ‘3’, and over two-thirds as ‘4’. Impacted teeth, non-odontogenic bone lesions, and condylar arthrosis were judged to be present (1) or absent (0). Maxillary sinuses were assessed for the presence (1) or absence (0) of a sinus pathology.

### Clinical data collection

Regarding clinical data collection, patients’ charts initially recorded at the appointment for oral focus evaluation were used. The pain percussion parameter was imported into a database for teeth with periapical lesions recorded on FDG-PET/CT (Excel, Microsoft), and the findings were noted as either 1 (parameter present) or 0 (parameter absent).

### Statistics

Ordinal non-dichotomous variables are expressed as median (range), and continuous variables were expressed as arithmetic mean ± standard deviation (SD). For periapical lesions and periodontal inflammation showing increased SUV_max_, radiological data (PAI and MPI scores) from oral radiographs were descriptively evaluated. Repeated measure correlation, including the repeated measure correlation coefficient (r_rm_), was tentatively conducted to examine the association between SUV_max_ and MPI score and between SUV_max_ and PAI score. The statistical significance level was set at *p* = 0.05. All statistical analyses and plots were performed using the statistical software program R [18], including tidyverse [19] and rmcorr [20].

## Results

### FDG-PET/CT results

Increased FDG uptake was recorded in 37 lesions in 18 of the 23 patients (78.3%). Of these, 12 were periapical lesions (mean SUV_max_ 6.1 ± 2.55), 15 were periodontal pockets (mean SUV_max_ 3.75 ± 1.03), and six were non-odontogenic bone lesions in the jaws (mean SUV_max_ 16.8 + /. ± 8.27); one was a retained tooth (SUV_max_ 13.8), two had tumours in the maxillary sinus (mean SUV_max_ 23.3 + /. ± 0.57), and one involved maxillary sinus membrane thickening (SUV_max_, 2.7).

### Periapical lesions

**Fig. 1:**
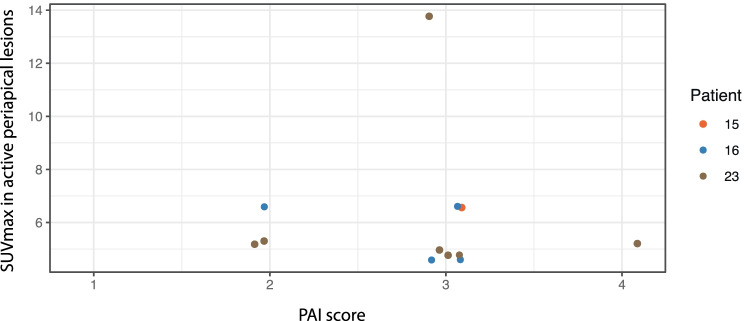
SUV_max_ in active periapical lesions in relation to PAI. Different colours indicate different patients

**Fig. 2:**
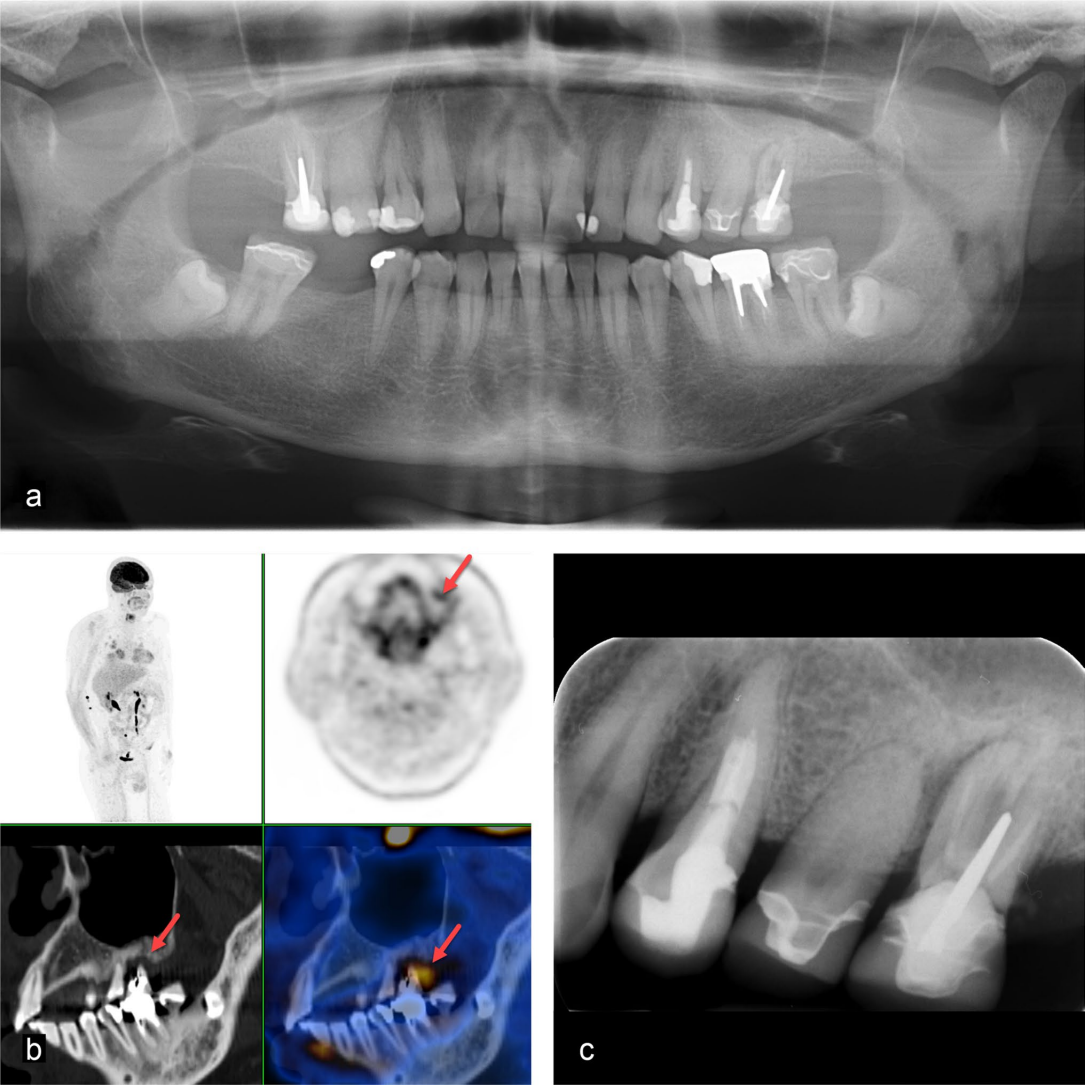
A 60-year-old woman with left-sided hypopharynx carcinoma cT2 cN0 cMx. Clinical examination showed increased mobility, probing depth, and a positive percussion test of tooth 26: **a** Panoramic radiography visualises impacted third molars in the mandible. **b** FDG-PET/CT shows a metabolically active osteolysis around tooth 26 with a SUV_max_ of 6.6 (red arrow). **c** The periapical radiograph visualises a combined perio-endo problem of tooth 26. The respective tooth was extracted, dental hygiene performed, and a fluoride splint prepared

**Fig. 3:**
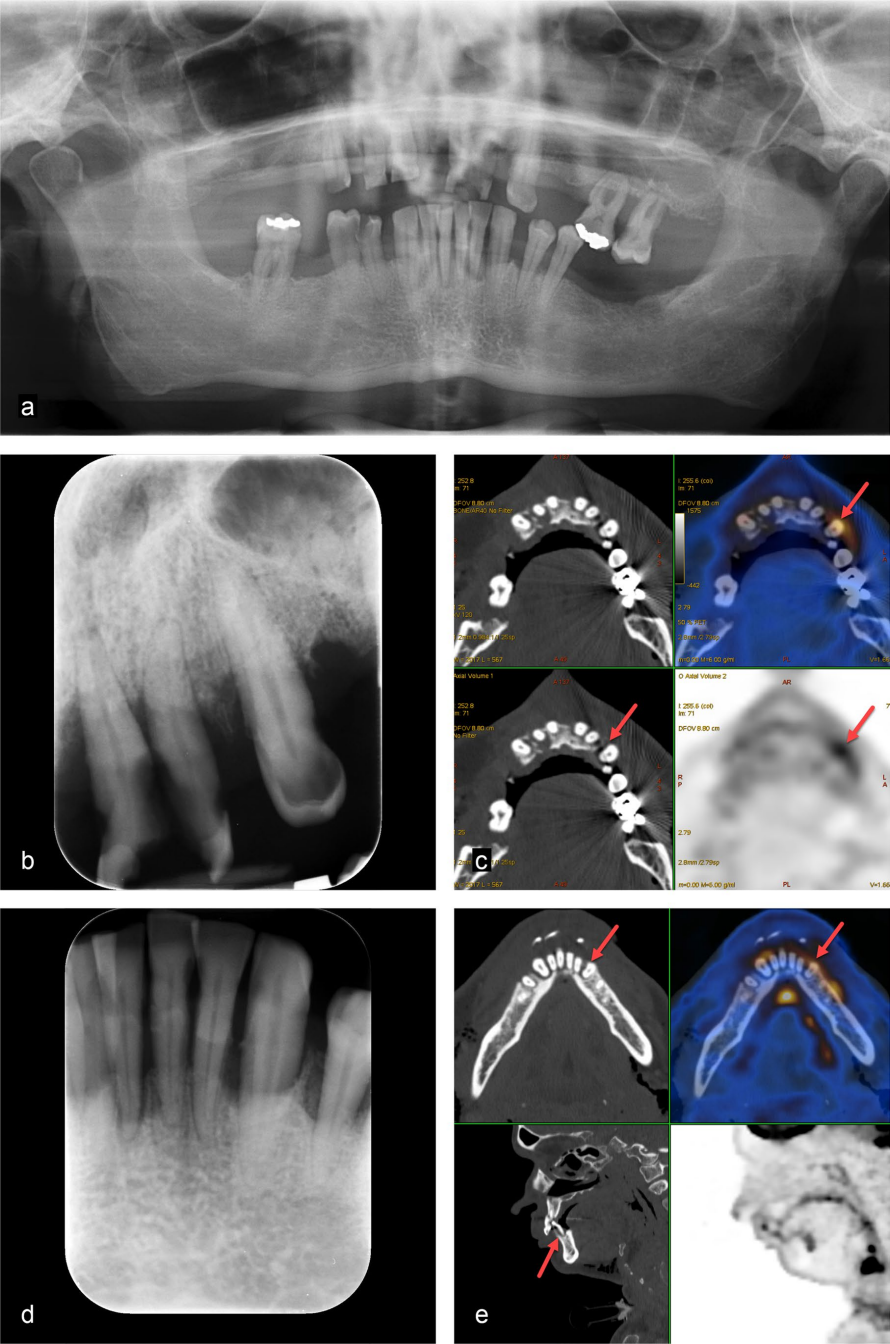
A 60-year-old man with glottic larynx carcinoma cT3 cN2c cM0. Clinical examination showed a generalised, severe chronic periodontitis and heavily decayed teeth due to caries and poor oral hygiene. Two out of several metabolically active pathologies are presented here in more detail. **a** Panoramic radiography gives an overview on the general periodontal situation. **b**, **d** The periapical radiographs of the upper left maxilla and the mandibular front show marginal periodontitis and vertical alveolar defects as well as tooth substance decay. **c**, **e** FDG-PET/CT demonstrates five tooth-related pathologies as metabolically active. The metabolic activity outlines the extent of the periodontal defects reaching to the apex for tooth 32 with a periapical periodontitis (SUV_max_ 6.6 for tooth 23 and SUV_max_ 4.6 for tooth 32, red arrows)

**Fig. 4:**
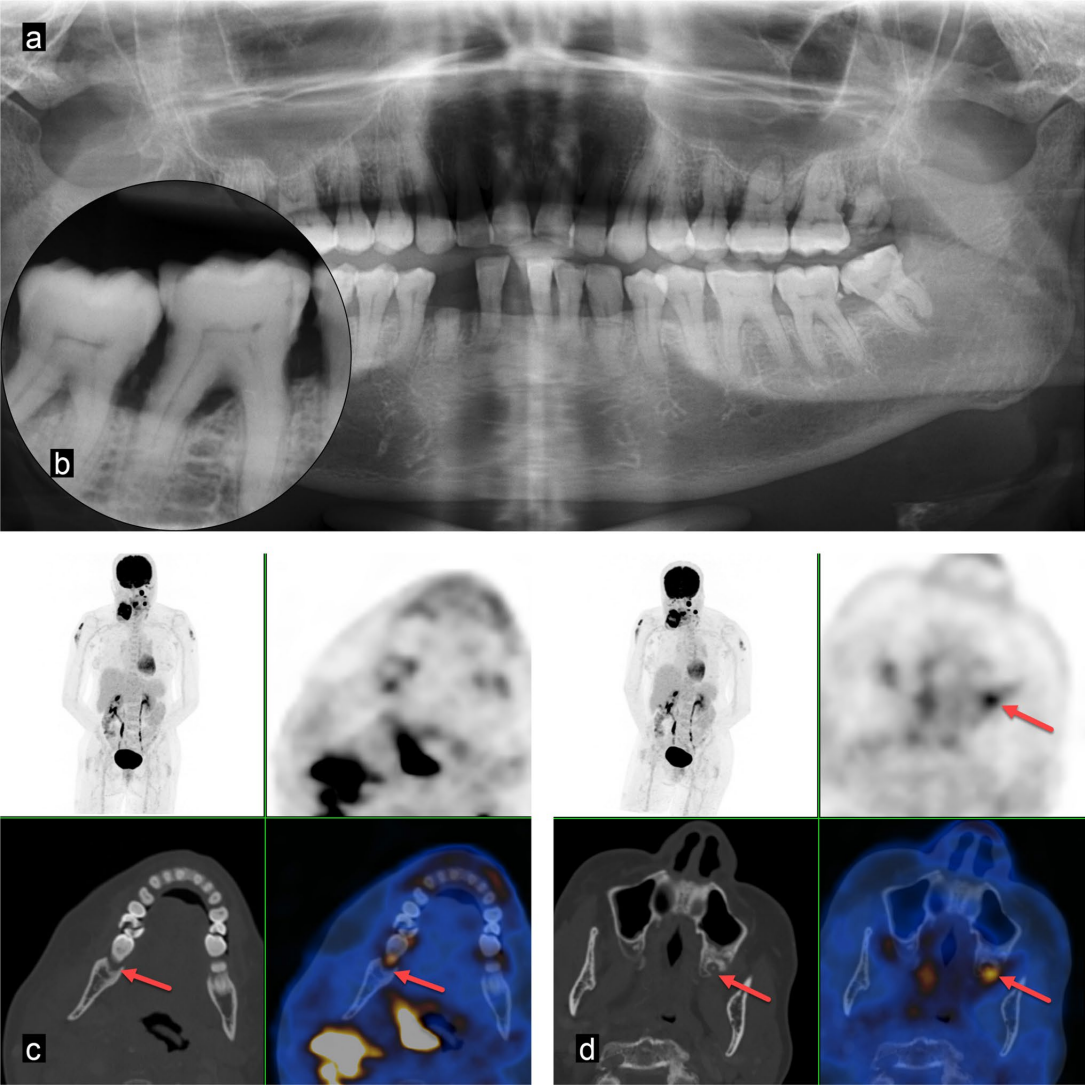
A 66-year-old woman with right-sided hypopharyngeal carcinoma cT4b cN2c M0. Clinical examination showed severe generalised chronic periodontitis and several heavily decayed teeth due to caries and poor oral hygiene. Two out of several metabolically active pathologies are displayed here. **a** Panoramic radiography shows several remaining tooth roots, calculus, and a reduced alveolar bone level. **b** The periapical radiograph of tooth 47 visualises a marginal periodontitis. **c**, **d** FDG-PET/CT demonstrates tooth-related pathologies with increased metabolic activity (SUV_max_ 13.8 for tooth 28 and SUV_max_ 5.0 for tooth 47, red arrows). All non-preservable teeth were extracted, preservable teeth restored, dental hygiene performed, and fluoride splints prepared

A total of 42 periapical radiotranslucencies were detected on all dental radiographs (OPT/periapical radiograph). A total of 50 periapical lesions were detected in 14 out of 23 patients (60.9%) on FDG-PET/CT. Hence, 16% of all periapical lesions cannot be detected on standard oral radiography. The median PAI score for these periapical lesions was 3 (range: 1–4). These periapical lesions were classified on the dental radiographs based on their PAI scores (Table 2). Eight additional lesions detected on FDG-PET/CT with a PAI score of 1 were not observed on the dental radiographs. Increased FDG uptake was observed in 12 out of 50 (24%) periapical lesions. The remaining 38 lesions did not show increased metabolic activity. The SUV_max_ of active lesions ranged from 4.6 to 13.8. Correlation analyses between SUV_max_ and PAI score were performed using repeated measure correlation and resulted in a correlation coefficient of r_rm_ = 0 (*p* = 1) (Fig. 1). Out of all periapical lesions, nine (18%) had previous root canal treatment, and one FDG-avid periapical lesion was found in an endodontically treated tooth (Fig. 2). The 12 FDG-avid lesions mentioned above were found in three out of 23 (13%) patients (Figs. 2, 3, 4). Case no. 3 showed a comparably high number (*n* = 14) of periapical lesions on the CT component of FDG-PET/CT, seven of which had increased FDG uptake. This patient did not report percussion pain in any of the teeth. Two out of the 12 PET-positive lesions (16.7%) had positive percussion test results. None of the patients without metabolically active periapical lesions showed a positive percussion test result.

**Table 2:** Periapical Index (PAI) distribution of periapical lesions (PAL)

PAI score	PAL (*n* = 50) (% of *n* = 50)	PAL with ↑ SUV_max_ (*n* = 12) (% of *n* = 12)
1	8 (16)	0
2	14 (28)	3 (25)
3	19 (38)	8 (67)
4	9 (18)	1 (8)
5	0	0

PAI distribution of 50 periapical lesions diagnosed on PET/CT in 14 out of 23 patients (61%)

### Marginal lesions

Out of the 418 teeth, 201 (48.1%) showed periodontal bone loss (vertical and/or horizontal) on dental radiography. These areas were classified according to MPI (Table 3). The median MPI score was 2 (range, 1–4). Out of the 201 periodontally affected areas, increased FDG uptake was recorded in 15 teeth (7.5%) and in eight out of 23 patients (34.8%). In other words, 186 areas did not show abnormal metabolic activity. SUV_max_ ranged from 2.5 to 5.7 (mean SUV_max_ 3.8).

**Table 3:** Marginal periodontal Index (MPI) distribution of teeth with marginal periodontitis (TMP)

MPI score	TMP (*n* = 201) (% of *n* = 201)	TMP with ↑ SUV_max_ (*n* = 15) (% of *n* = 15)
1	78 (39)	0
2	61 (30)	0
3	41 (20)	4 (27)
4	21 (11)	11 (73)

MPI distribution of 201 periodontally compromised teeth out of 418 analysed teeth (48%)

**Fig. 5:**
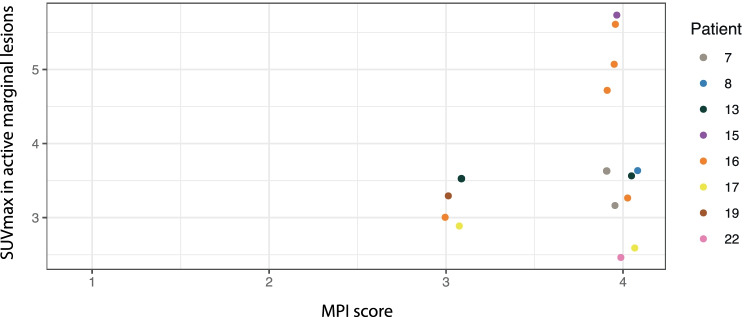
SUV_max_ in active marginal periodontitis in relation to MPI. Different colours indicate different patients

The correlation analysis between SUV_max_ and MPI score was performed using repeated measure correlation and yielded a correlation coefficient of *r*_rm_ = 0.4 (*p* = 0.32) (Fig. 5).

### Maxillo-mandibular structures and inflammatory pathologies

The analysis of the available radiological data in this study revealed several possible inflammatory conditions in the jaws of the included patients. Table 4 shows the number of all pathologies detected and the number of pathologies with increased FDG uptake. Out of the 46 mandibular condyles in 23 patients, eight showed signs of osteoarthritis on FDG-PET/CT, but without increased FDG uptake. Out of the 46 maxillary sinuses, 20 showed sinus pathology, including swelling of the basal membrane (*n* = 7), single or multiple cysts (*n* = 9), both membrane swelling and mucus retention cysts (*n* = 2), and tumour infiltration (*n* = 2). Three maxillary sinuses showed increased FDG uptake, which clinically corresponded to squamous cell carcinoma in two cases and swelling of the basal sinus membrane in one case. Non-odontogenic bone lesions were caused by osseous tumour infiltration in 6 out of 23 (26.1%) patients, causing FDG uptake. Two cases were located in the left mandible, three in the right mandible, and one in the right maxilla connected to the sinus. All tumours were histopathologically verified as squamous cell carcinomas. One partially erupted wisdom tooth (Fig. 4) showed an increased FDG uptake.

**Table 4:** Maxillo-mandibular structures and inflammatory pathologies

Maxillo-mandibular pathology	Patients with pathology (*n* = 23) (% of *n* = 23)	Total sites/pathological sites	Sites with ↑ SUV_max_	Comment on sites with ↑ SUV_max_	SUV_max_ range (mean SUV_max_ ± SD)
Mandibular condyle pathology	5 (22)	46/8 Condyles	0	-	-
Maxillary sinus pathology	14 (61)	46/20 Sinuses	3	1 × basal membrane swelling 2 × tumour infiltration	2.7–23.7 (23.3 ± 0.57)
Non-dentogenous bone lesion	6 (26)	92/6 Jaw quadrants	6	6 × tumour infiltration	8.0–29.1 (16.8 ± 8.30)
Retained tooth	5 (22)	418/7 Teeth	1	Wisdom tooth	13.8

## Discussion

The aims of this study were to analyse the distribution and metabolic activity of periapical radiolucencies, marginal periodontitis, and possibly inflammatory maxillo-mandibular pathologies on FDG-PET/CT scans and to compare these with their appearance on standard oral radiographs. The results showed that a large percentage of all radiologically detected periapical lesions, areas of marginal periodontal bone loss, impacted teeth, and other possible inflammatory pathologies of the jaws did not show increased metabolic activity or other signs of acute inflammation on FDG-PET/CT, regardless of their radiological extent or location.

Due to the small sample size of the retrospectively examined data, neither a correlation between FDG uptake and the PAI score nor between FDG uptake and the MPI score could be statistically demonstrated. Although FDG uptake and PAI score seemed unlikely to be associated in our study (*r*_rm_ = 0; *p* = 1), a relationship between FDG uptake and MPI score was deemed possible according to the moderate correlation found in our data (*r*_rm_ = 0.4; *p* = 0.32).

### Comparison with existing literature

FDG accumulation in marginal and periapical lesions has been analysed in a comparable study [17]. Metabolic activity (as measured by SUV_max_) was compared to the extent of marginal bone resorption and showed a moderate correlation. Regarding marginal periodontitis, the SUV_max_ tended to be higher in teeth with higher marginal bone loss. These findings were consistent with our results. Furthermore, the authors found a moderate correlation between SUV_max_ and periapical radiolucent area size. Our study did not assess the SUV_max_ of all tooth apices but only those showing increased FDG uptake on PET/CT. These signs of inflammation were detected in 12 periapical lesions. Interestingly, SUV_max_ was not associated with the PAI score. A limitation of this retrospective study is that the standard oral radiography is influenced by the applied short cone, leading to magnification. Furthermore, projectional angulations on the sensor in periapical radiographs could differ owing to the applied technique, depending on the alveolar position of the teeth. The main emphasis of this study was the analysis of the metabolic activity of radiological findings detected on PET/CT and oral radiographs. Thus, the study was limited to a comparison of these methods and the clinical interpretation of the corresponding data.

Yamashiro et al. reported increased FDG uptake in acute periodontal disease, acute apical periodontitis, and pericoronitis of third molars [21]. The authors also detected an association between FDG uptake and radiological degree of inflammation. Similar to the results of the present study, FDG did not accumulate in chronic apical infections. The authors stated that an FDG-PET/CT scan may serve as a reliable preoperative tool to identify high-risk patients with acute oral infections who must be referred to oral care specialists.

Since this study was a retrospective analysis, clinical parameters such as probing depth, tooth mobility, bleeding on probing, and oral hygiene condition could not be obtained for all teeth. This is a limitation of this study. Percussion pain served as the sole parameter used to assess the acute state of infection. Only two out of 12 FDG-avid periapical lesions showed pain on percussion. Thus, FDG uptake and percussion pain were not associated with each other. FDG uptake may also occur in acute infective exacerbations of chronic inflammatory processes of the jaw, such as osteonecrosis [22, 23]. Various studies have assessed the short-term and long-term oral complications of radiochemotherapy in patients with HNC and the rationale for focus assessment prior to cancer therapy [8, 24, 25]. Thorough dental examinations, patient counselling, and extraction of highly problematic teeth before radiotherapy are strategies adopted by most head and neck tumour centres [26]. However, the rate of severe complications, such as osteoradionecrosis, remains significant (up to 5%) [27, 28]. Early detection of FDG-avid inflammatory foci on staging FDG-PET/CT, which is conducted early after cancer diagnosis, may help further reduce such events. In addition, FDG-PET/CT is an established diagnostic tool for reviewing head and neck cancer after radiotherapy [29]; therefore, radiation-induced dental and jaw pathology might also be detected using this modality.

Some authors consider leaving untreated chronic foci in patients undergoing intensive chemotherapy [30], without observing any increase in infective complications. This could be related to the low rate of pain on percussions noted in the present study. Most of the periapical lesions detected did not show increased FDG uptake and were considered chronic rather than acute. However, another study by the same research group showed that untreated patients scheduled for IMRT with severely periodontally compromised teeth and subsequent extractions were more prone to develop ORN [31]. Our results show that increased FDG uptake is mainly seen in advanced marginal periodontitis (MPI score 3–4), which aligns with the results of the abovementioned study. For this reason, metabolic information, such as FDG uptake, might guide decision-making prior to radiotherapy or chemotherapy. Similarly, a recent review analysed the usefulness of defining oral foci (acute and chronic) with regard to possible elimination before oncological treatment [32]. Chronic oral foci could be surveyed in chemotherapy patients and, if needed, be treated in the remission phase. Asymptomatic lesions should be treated conservatively to avoid ORN induction.

For patients undergoing radiation therapy at the present study centre, oral care was performed using a standardised protocol to mitigate oral mucositis and its consequences. During 6 weeks of radiation therapy, the patients received fluoride-filled splints. The splints were applied during radiation therapy as well as in the morning for 5 min using a PlakOut Gel (KerrHawe SA, Bioggio, CH) and 5 min in the evening using Duraphat (Colgate, Therwil, CH). Before splint application, patients brush their teeth and rinse their mouths. During radiotherapy, a dental hygienist evaluates and monitors oral care once a week. In addition to the awareness of oral care specialists regarding the importance of avoiding inflammation during radiotherapy, nuclear medicine physicians and radiologists should be aware of the importance of oral focus treatment. They should promptly report to an oral care specialist if the FDG-PET/CT scan shows evidence of increased inflammatory activity [8]. On the other hand, oral care specialists should be aware that useful metabolic information derived from FDG-PET/CT might be available to assist with oral disease diagnosis in their patients. A recent FDG-PET/CT scan may help determine the activity of an oral disease focus and inform decision-making regarding which to treat and which to observe.

## Conclusion

FDG-PET/CT provides additional metabolic information to clinicians, especially through the detection of acute inflammation in periapical radiolucencies, marginal periodontitis, and possibly inflammatory maxillo-mandibular pathologies. Combining all the available imaging information may lead to a more accurate diagnosis of oral focal lesions and enable appropriate treatment.
